# Fur Color and Nutritional Status Predict Hair Cortisol Concentrations of Dogs in Nicaragua

**DOI:** 10.3389/fvets.2020.565346

**Published:** 2020-10-19

**Authors:** Grace B. Bowland, Robin M. Bernstein, Jeremy Koster, Christine Fiorello, Maris Brenn-White, James Liu, Laura Schwartz, Amanda Campbell, Devin von Stade, Janet Beagley, Julie Pomerantz, Alejandro González, Mackenzie Quick, Kailyn McKinnon, Andrea Aghaian, Corey Sparks, Joshua B. Gross

**Affiliations:** ^1^Anthropology, University of Colorado Boulder, Boulder, CO, United States; ^2^Institute of Behavioral Science, University of Colorado Boulder, Boulder, CO, United States; ^3^Department of Anthropology, University of Cincinnati, Cincinnati, OH, United States; ^4^Department of Human Behavior, Ecology, and Culture, Max Planck Institute for Evolutionary Anthropology, Leipzig, Germany; ^5^University of California, Davis, Davis, CA, United States; ^6^Saint Louis Zoo, St. Louis, MO, United States; ^7^Microbiology, Immunology, and Pathology, Colorado State University, Fort Collins, CO, United States; ^8^Independent Researcher, Seattle, WA, United States; ^9^Independent Researcher, Castalia, NC, United States; ^10^Independent Researcher, Veracruz, Mexico; ^11^Department of Demography, University of Texas at San Antonio, San Antonio, TX, United States; ^12^Department of Biological Sciences, University of Cincinnati, Cincinnati, OH, United States

**Keywords:** hair cortisol concentrations, canine health, canine nutrition, cortisol and metabolism, stress energetics in working dogs, hair pigmentation

## Abstract

This study examined the relationships between hair cortisol concentrations (HCC) and sex, age, nutritional status (as determined by body condition scores, or BCS), and body mass (geometric mean calculated from morphometric measurements), as well as the potential influence of hair pigmentation (light, dark, or agouti/mixed) on HCC in dogs of the Bosawas Biosphere Reserve, Nicaragua. The dogs examined in this study live in a marginal environment where disease, malnutrition, and mortality rates are high. For fur color, HCC was significantly higher in light fur than in than dark and mixed fur (*p* < 0.001). In addition, BCS scores were found to have a negative effect on HCC (*p* < 0.001). Measures of sex and body size exhibited inconclusive effects on HCC, and when compared to adult dogs, juvenile dogs did not exhibit significantly different HCC. Repeated measures of dogs over time reveal a moderate intra-class correlation, suggesting that there are unmeasured sources of individual-level heterogeneity. These findings imply a need to account for fur color in studies of HCC in dogs, and the study suggests an overlooked relationship between cortisol and body condition scores in undernourished dogs in diverse settings.

## Introduction

Cortisol is a glucocorticoid hormone produced by the neuroendocrine pathways of the hypothalamic-pituitary-adrenal (HPA) axis in response to changes in basal homeostasis. In many mammals, cortisol is responsible for maintaining many of the body's day-to-day and long-term functions, including metabolism, immune system function, growth and reproduction, and sleep/wake cycles (1–4). Cortisol plays a critical role in the body's stress response and increases in cortisol are an adaptive response to aid the body in handling physical or perceived environmental stressors. In the short-term, this response is beneficial. However, long-term cortisol elevation is maladaptive and has been implicated in immune suppression and stunting of growth and development (2–4). Due to the integral role of cortisol to overall health and behavior, analysis of cortisol is often used when assessing the effects of potential stressors (which may range in degree of severity) such as malnutrition, illness, and physical or psychological trauma (5). For both working and companion dogs, cortisol analysis can provide vital insight into physiological health and behavior and allow greater insight into how our canine counterparts adapt to their environment and human-directed tasks.

Studies on the impact of environmental factors such as home life, seasonality, weather, and human-dog interactions on dogs have been conducted through cortisol analysis (6–14). Research has also been conducted in dogs to assess the effect of certain activities such as playing and socializing on cortisol levels, and further research has explored the link between cortisol and lifestyles in working and companion dogs (10, 12, 15).

Associations between cortisol and phenotypic factors, such as body mass, metabolic rate, sex, and age have been explored in domestic canines as well as other mammals. For instance, in a study of 48 German Shepherd and Labrador Retrievers, Sandri et al. (12) found that salivary cortisol concentrations in dogs were inversely related to size. Gillooly et al. (5) confirmed that this trend appears to hold across cortisol-dominant mammal species. Size, defined as body mass, and cortisol have both been related to metabolic rate in mammals. Cortisol plays a regulatory role in metabolic rate, which also decreases with mass (5). In addition, sex-based differences in cortisol levels have been noted in several mammalian species, though these findings have been less consistent. In humans, males tend to have higher cortisol levels on average than females (16) whereas in vervet monkeys this pattern was observed to differ, with males exhibiting lower cortisol levels than females (17). In some cases, research on dogs has failed to find any significant difference in cortisol by sex (11). However, whereas Sandri et al. (12) noted no difference between intact male and female dogs, castrated males and spayed females both showed significantly lower cortisol levels than their intact counterparts. Overall, these studies reveal substantial heterogeneity by sex—both across and within species—which suggests that further research on sex-based differences in cortisol is merited.

A study of salivary cortisol in canines revealed age-related differences in cortisol wherein younger dogs (< 6 months) have significantly lower cortisol levels than their older counterparts (18). Interestingly, these findings conflict with the results of another salivary cortisol study in Dingos, which found puppies had significantly higher cortisol levels than adults (19). The incongruent findings from these studies suggests the potential for developmental differences in cortisol levels in canines, but further research is necessary to elucidate the effects of age.

In addition to size and metabolic rate, nutritional health has also been closely related to cortisol levels in humans and some wild mammals (20, 21). A widely practiced method among veterinarians of assessing body composition in dogs is the use of body condition scoring (22). While often used in industrial settings to assess the presence of excess fat, and the potential risk for a dog to become obese, it can also be used to evaluate the risk or presence of underweight status. Ranging from a minimum value of 1 for malnourished dogs to a maximum value of 9 for obese dogs, veterinarians consider a body condition score (BCS) of 4–5 to be ideal, while values outside this range are potentially worrisome (23). At present, we are unaware of any studies which have explored the relationship between cortisol and BCS scores among dogs.

Hair provides both a non-invasive and reliable method for measuring time-averaged cortisol levels, as the bioavailable (or unbound) fraction of the hormone is incorporated into the hair shaft during hair growth (24). Hair cortisol analysis has been conducted in a wide range of species, including a number of both wild and domesticated animals such as dogs, to assess cortisol variation in relation to stress, growth, and development (6, 11, 12, 24–28). By providing a time-averaged measure, hair samples are distinguished from other sample types, including saliva, blood, and feces, which largely reflect short-term fluctuations in cortisol (29).

The measurement of hair cortisol in domesticated dogs has been validated by numerous studies (9, 24, 30). This body of research suggests that hair pigmentation is a potential confounding factor that should be considered when investigating other predictors. For example, sable fur has been shown to retain significantly lower concentrations of cortisol when compared to yellow, white, or red fur, while agouti fur did not significantly differ from either the dark or light coat coloring (30). The reasons for differences in cortisol levels of differently pigmented hair is not clearly understood but may relate to the physical size of different melanin granules (30).

This paper presents results from hair cortisol analysis of hunting dogs from the indigenous Miskito and Mayangna communities living in the Bosawas Biosphere Reserve of Nicaragua. This population provides a unique opportunity to inform and expand current literature on hair cortisol analysis in dogs and stress energetics. Current literature on the subject of stress in dogs is based on studies with small sample sizes in highly regulated clinical settings in North America or Europe. Furthermore, the dogs included in these studies are primarily purebred. In contrast to these populations, many dogs around the world live in environments with inadequate nutrition and veterinary care (31–33). Little is known about the energetics of stress in these contexts, which are arguably more relevant for understanding the settings in which domestic dogs first evolved. Previous studies in the Bosawas Reserve and analogous settings show that high rates of disease, malnutrition, and injury contribute to high mortality among dogs (34–36). The contrast with purebred dogs in high-income countries is noteworthy (37).

The objectives of this study were to test for the effects of fur color, sex, age, body size, and nutritional status on hair cortisol to assess the robustness of predictions in this population of village dogs from Nicaragua.

## Methods

### Ethics Statement

The study was reviewed and approved by the Institutional Animal Care and Use Committee of University of California, Davis. Informed consent was verbally obtained from all of the human participants, as many of the dog owners were functionally illiterate.

### Study Site

**Figure 1:**
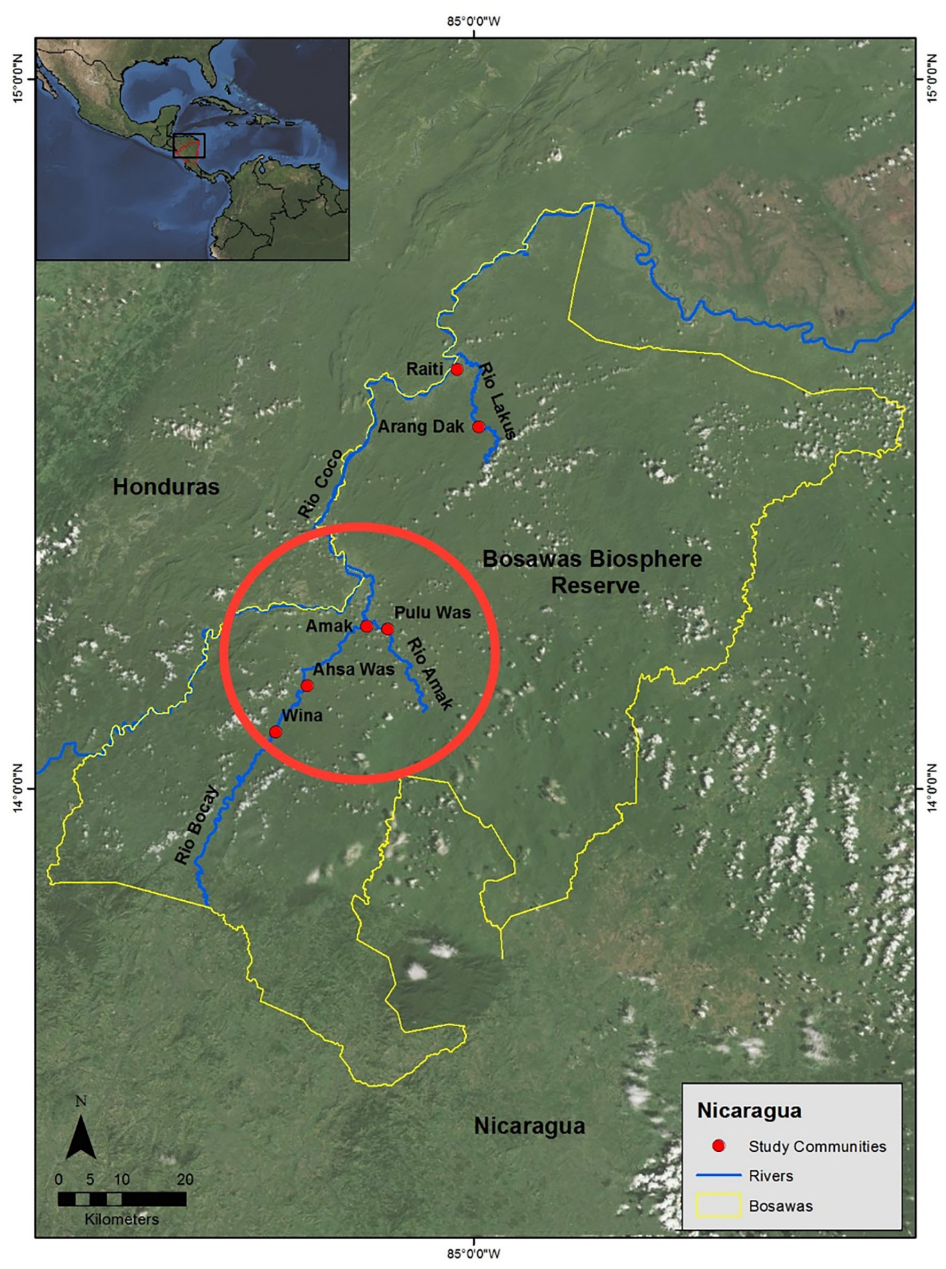
Map of the Bosawas Biosphere Reserve. The study communities of Amak, Pulu Was, Ahsa Was, and Wina are circled in red.

This study was conducted among the indigenous Mayangna and Miskito residents of the Bosawas Biosphere Reserve in Nicaragua (Figure 1). The reserve is characterized by a tropical rain forest biome and harbors a diverse flora and fauna (38–40). The Mayangna and Miskito, who compose the country's most predominant indigenous groups, rely on subsistence practices such as swidden horticulture for most of their nutritional needs (41, 42). Cultivated crops include rice, beans, manioc, and bananas. The Mayangna and Miskito also maintain domestic animals, such as chickens, cattle, and pigs, and they supplement their diets with hunting and fishing, which are the primary sources of dietary protein (43).

Dogs in the Bosawas Reserve are owned by families, who assign them names and provision them with food. The dogs are fed portions of the same foods prepared for their households, and stable isotope analysis suggests that the composition of their diets is largely comparable to the human diet (44). At night, dogs typically sleep in the house, often in the cooking area. Puppies receive affectionate attention, but owners rarely pet their adult dogs, and human-dog relationships differ from the norms in Western contexts (45, 46). Dogs are typically unrestrained and can walk around the community during the day. However, dogs sometimes arouse antagonistic treatment from others in the community, and dogs are seemingly wary of physical abuse when they tread into unfamiliar parts of the community. Notably, owners rarely bathe their dogs, which mitigates concerns that their hair cortisol concentrations are impacted by shampoos or hot water (47, 48).

Dogs are commonly used as hunting companions, and ~85% of harvested mammals are captured with the assistance of dogs (49). However, there is high heterogeneity in the hunting performance of dogs, and many of the dogs provide little value as hunting companions (50). Veterinary evaluations of the health status of dogs at Bosawas have indicated that malnutrition and dehydration are common in this population (35). Serosurveys also suggest high rates of canine distemper virus, canine parvovirus, *Rickettsia rickettsii*, and *Leptospira* spp (35). The elevated prevalence of disease and injury during hunting contribute to a high mortality rate, and few dogs live beyond 6 years old (35, 43). Dogs within these communities are not spayed or neutered, and routine veterinary care is virtually non-existent. Owners may treat their dogs with antiparasitic medications (e.g., Ivermectin) or antibiotics (e.g., Oxytetracycline) when they suspect infections, but the use of such medications tends to be sporadic. Local residents report that government teams periodically circulate throughout the reserve to administer vaccinations for rabies. See Supplementary Figures 1–3 for photographs of three dogs in the sample, which provide examples of the categorization of fur color used in this study.

### Data Collection

A group of veterinarians examined each dog, documenting variables such as general physical and behavioral attributes, vital measurements, sex, and appearance of health, including any medical problems present. To determine the size of the dogs, morphometric measurements were obtained for height, body length, chest-width, chest-girth, and head-width (51). In assessing the nutritional status of each dog, a numerical value was assigned using the Body Condition System (BCS) scale of 1–9 (Supplementary Figure 4). The owner reports of the dogs' ages were subject to measurement error, so as a preliminary measure of age, the data collection team checked dental eruption patterns and noted which dogs appeared to be juveniles (36). Employing a threshold of ~6 months, we use a binary variable to distinguish the mature dogs from the juveniles in the sample.

Hair samples were also obtained during examination by cutting a small chunk of fur from the coat using scissors or shears. Hair samples were taken from the same location on each dog, specifically the dorsal base of the tail.

Annual exams were conducted during the months of July and August in three field seasons from 2014 through 2016. During this time, ~750 hair samples were collected, from a total of 580 unique dogs from the communities of Amak, Pulu Was, Ahsa Was, and Wina. Due to occasional errors in labeling of hair samples, insufficient sample weights, and sample loss during processing, seventy-nine samples (~10%) were unusable (final study *N* = 672, 454 unique individuals). Of the total population of sampled dogs in this study, 213 were female and 240 were male (the sex of one individual was not recorded). When possible, the team attempted to examine and collect hair samples from the same dogs during each of their visits, thus providing a set of longitudinal data on these subjects. However, some dogs were not present for multiple examinations because of mortality or relocations outside the study communities. Of the 454 unique dogs in the sample, 118 dogs contributed two measurements to the compiled sample and 50 dogs contributed three measurements to the compiled sample.

### Dog Hair Cortisol Processing

Upon collection, each hair sample was placed in a paper envelope labeled with an identifying number and the date of exam. The samples were then shipped to the Growth and Development Laboratory at the University of Colorado, Boulder for processing and analysis. Samples ranging from 10 to 30 mg of hair were weighed and placed into 2 mL Eppendorf tubes. Weights were recorded along with the corresponding ID number and date, as well as the fur color. Fur color was denoted as either dark (D), mixed (M), or light (L), and categories were defined according to the parameters used by Bennett and Hayssen (30). Each hair sample was then washed a minimum of three times with 1.5 mL isopropanol to ensure all dirt, dead skin, and sebum were removed. Samples were left to air dry under a fume hood for ~2 days to ensure complete evaporation of the isopropanol. The hair was ground by placing a stainless steel or tungsten carbide ball in each tube and using a Ball Mill at 25 Hz for 10 min. Samples where hair was not sufficiently ground were placed back on the machine and ground for an additional 5 min. After grinding, 1 mL of methanol was added, and samples were vortexed before being placed on a shaker plate (~100 rpm) at room temperature to extract overnight. The following day, samples were centrifuged for 12 min at 2,500 rpm. Subsequently, 850 uL of supernatant was extracted and transferred to a clean Eppendorf tube. The supernatant was then evaporated using a Microvap nitrogen dry-down evaporator (Organomation, Berlin MA) with tubes in a heated (63°C) block for ~16 min. If at the end of the drying time some liquid was still present, samples were left for a few more minutes. Samples were then reconstituted using 0.2 mL buffer solution (Salimetrics, Carlsbad CA) and stored at −20°C until processing.

Hair cortisol concentrations were quantified using enzyme linked immunosorbent assay (ELISA- Salimetrics, Carlsbad CA). The use of the Salimetrics kit for canine hair cortisol analysis has been validated by previous studies (8, 30). Results were converted from ng/mL to pg/mg for statistical analysis.

### Analysis

HCC was log10 transformed because of its positively skewed distribution. Plots are displayed on the log-transformed scale, though the supplemental material displays plots on the untransformed scale (Supplementary Figures 5–7).

As a measure of the overall size of individual dogs, the geometric mean of the combined height, body length, chest width, chest girth, and head width measurement data was calculated^1^.

Our analytical strategy involved the specification of multiple regression models, which provide insights into the variance components of the data and the bivariate effects of the covariates. These covariates include fur color, sex, age, body condition scores, and body size (i.e., the aforementioned geometric mean).

Owing to the repeated measure of dogs across field seasons, all models included a random effect (a varying intercept) for the individual dogs. The variance estimates of these random effects can be compared to the residual variance to calculate the intra-class correlation (52, 53). The intra-class correlation, which is the ratio of the random effect variance to the total variance, is interpretable as the expected correlation between successive measurements of the same unique dogs. In addition to the other regression models, we include an “empty” model that has no fixed effect covariates other than the intercept in order to obtain an estimate of the intra-class correlation that is not adjusted for the predictors.

The final model includes all of the covariates, which are expected to exhibit additive effects on HCC.

All analyses were performed using JMP Pro 14 software.

## Results

Descriptive statistics are reported in Table 1. Results of the statistical analyses are reported in Table 2.

**Table 1:** Descriptive statistics.

**Variable**	**Description**	***N***	**Mean**	**Std. Dev**.	**Min**	**Max**
Cortisol (pg/mg)		672	17.87	9.62	6.1	121.2
Body Condition Score (BCS)	On a scale of 1–9	663	2.3	0.88	1	6
Size (Geometric Mean) (cm)	Composite of height, body length, head width, chest width, chest girth	579	57.3	7.3	25.1	78.1
**Variable**	**Description**	***N***	**Proportion**			
**Fur Color**		672				
L	Light–yellow, red, or white		0.36			
M	Mixed or agouti		0.27			
D	Dark or Sable		0.36			
**Sex**		672				
Female	Female		0.47			
Male	Male		0.53			
**Age**		663				
Juvenile	>6 months		0.08			
Adult	<6 months		0.92			

*Of the dogs in the sample, 118 dogs contributed two measurements of hair cortisol to the compiled sample, and 50 separate dogs contributed three measurements to the sample. Categorical variables are reported as proportions of the total (owing to rounding error, the proportions for fur color do not sum to 1)*.

**Table 2:** Multilevel regression model results where hair cortisol [log10(pg/mg)] is the dependent variable, and fur color, sex, body condition score, geometric mean, and a binary measure of age are the independent variables.

**Parameter**	**Model 0**	**Model 1**	**Model 2**	**Model 3**	**Model 4**	**Model 5**	**Model 6**
Fur (Mixed)		−0.070 (0.015)^***^					−0.068 (0.016)^***^
Fur (Dark)		−0.075 (0.014)^***^					−0.071 (0.016)^***^
Female			0.027 (0.013)^*^				0.014 (0.014)
Age (Adult)				−0.034 (0.021)			−0.014 (0.032)
BCS					−0.03 (0.007)^***^		−0.03 (0.007)^***^
Geometric Mean						−0.005 (0.002)^**^	−0.002 (0.002)
Constant	1.222 (0.006)^***^	1.268 (0.01)^***^	1.21 (0.009)^***^	1.253 (0.02)^***^	1.29 (0.017)^***^	1.35 (0.042)^***^	1.397 (0.052)^***^
Dog ID Variance	0.006 (0.002)^**^	0.005 (0.002)^**^	0.006 (0.002)^**^	0.006 (0.002)^**^	0.006 (0.002)^**^	0.006 (0.002)^**^	0.004 (0.002)^*^
Residual Variance	0.017 (0.002)	0.017 (0.002)	0.017 (0.002)	0.017 (0.002)	0.017 (0.002)	0.017 (0.002)	0.018 (0.002)
Intra-class correlation	0.27	0.22	0.26	0.27	0.26	0.26	0.20
Observations	672	672	671	663	663	579	566

*Standard errors are reported in parentheses*.

*, **, *** *indicates significance at p < 0.05, p < 0.01, and p < 0.001, respectively*.

*All models included a varying intercept for individual dogs, some of which were sampled multiple times throughout the study*.

Model 0 is the empty model, which reveals an intra-class correlation of 0.27. This correlation is the expected correlation between successive measurements of the same dogs over time (with samples collected ~1 year apart). Although somewhat modest, the correlation remains largely consistent across all models, which suggests that the correlation stems from unmeasured sources of individual-level heterogeneity.

**Figure 2:**
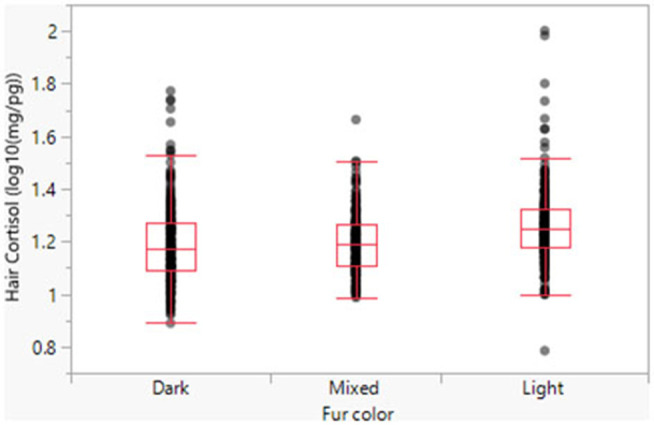
Hair cortisol concentrations as a function of fur color.

Model 1 suggests a significant relationship between fur color and HCC, with dark and mixed fur exhibiting significantly lower cortisol levels than light fur (Figure 2). When back-transformed, the respective predicted means are 18.53 pg/mg (light fur), 15.78 pg/mg (mixed fur), and 15.60 pg/mg (dark fur).

**Figure 3:**
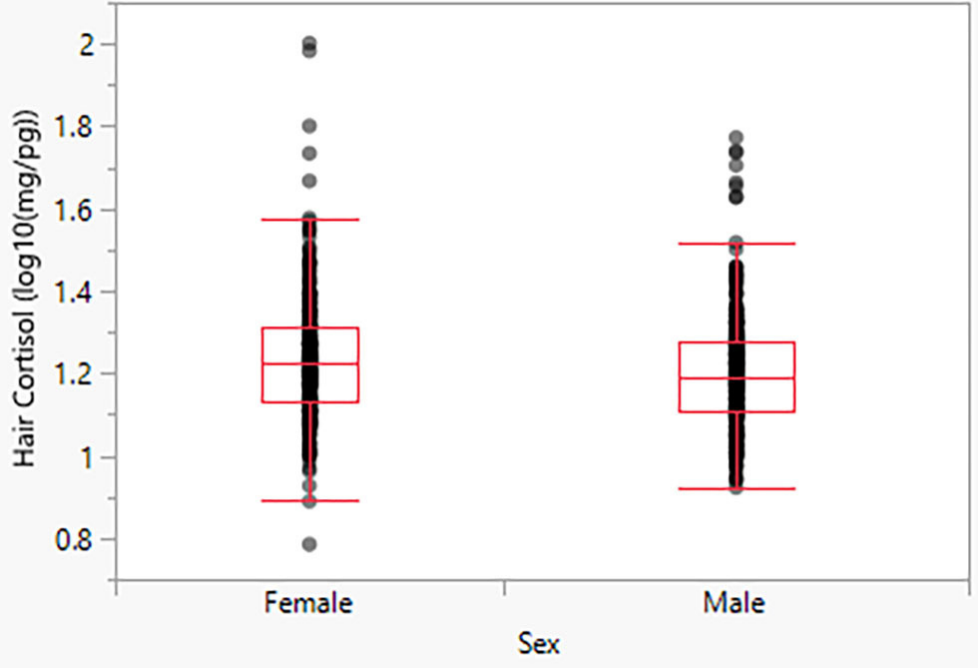
Hair cortisol concentrations as a function of sex.

Model 2 indicates a significant effect of sex, with females exhibiting moderately higher HCC than males (*p* < 0.05). The predicted mean for males is 16.22 pg/mg, and the predicted mean for females is 17.26 pg/mg (Figure 3).

Model 3 does not reveal a significant effect for the binary measurement of age (β = −0.034). This result suggests that juvenile and adult dogs in this population have roughly comparable hair cortisol concentrations.

**Figure 4:**
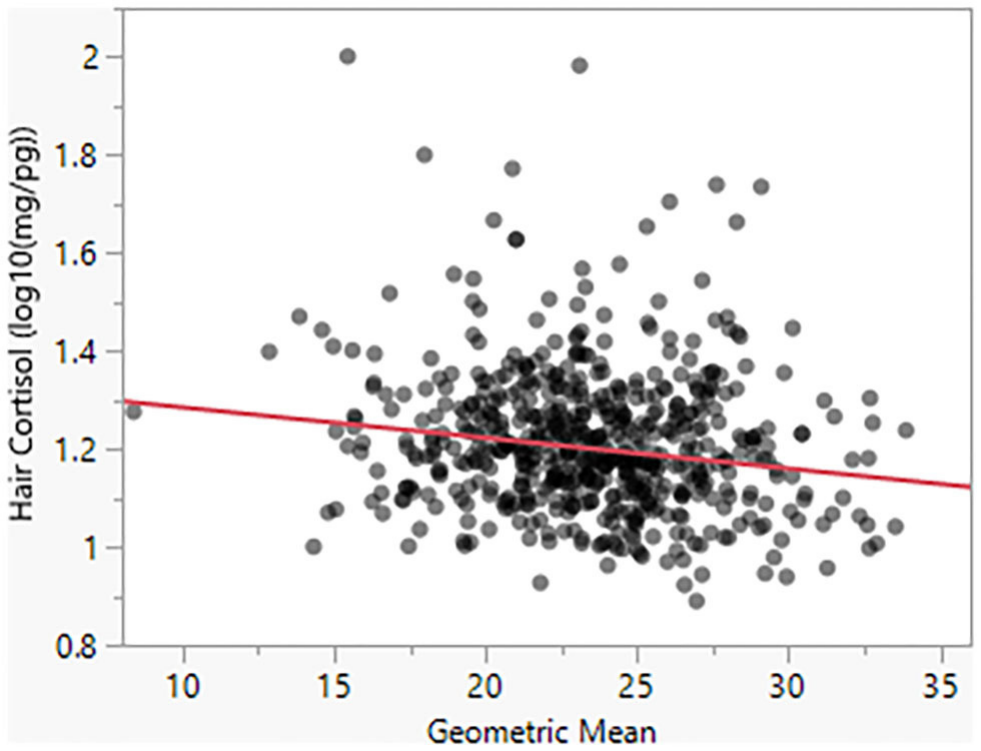
Hair cortisol concentrations as a function of body condition scores.

**Figure 5:**
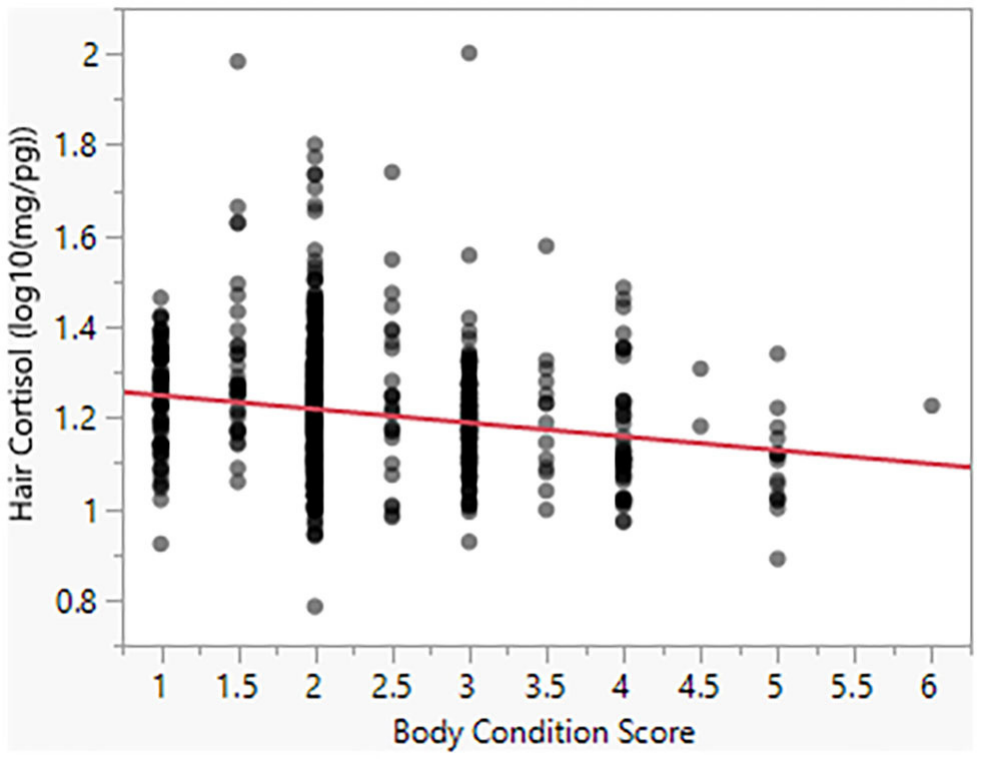
Hair cortisol concentrations as a function of body size, as reflected in the geometric mean of morphometric measurements.

Model 4 suggests that Body Condition Score has a significant negative effect on HCC (β = −0.03). For a BCS value of 1, the model predicts HCC of 18.20 mg/pg, as compared to a predicted value of 13.80 for a BCS value of 5 (Figure 4). Similarly, as seen in Model 5, body size has a negative effect (β = −0.005) on HCC (Figure 5).

In Model 6, which includes all of the predictors, only fur color and BCS continued to exhibit significant effects. Because male dogs are larger than females, it may be that sex and body size exhibit collinearity that reduces the predicted effects of the respective coefficients.

## Discussion

In this study of hair cortisol concentrations of hunting dogs living in Nicaragua's Bosawas Biosphere Reserve, the effect of hair pigmentation was found to be statistically significant on reported cortisol levels. Light fur color was associated with significantly higher cortisol concentrations than both dark and mixed fur. In addition, body condition exhibited a negative correlation with HCC. While a bivariate model initially indicated that female dogs show higher cortisol levels than males, sex was not found to be a significant factor in the model including the full set of covariates. Similarly, geometric mean as an indicator of body size shows a negative correlation with cortisol concentrations that is not evident in the model that includes other predictors.

The findings for fur color expand on the previous findings of Bennett and Hayssen (30), which suggested that different hair colors sequester cortisol heterogeneously. We did not attempt to replicate their test of different hair colors from the same dog. Our results, however, further imply that fur color should be a control variable in analyses that use hair samples to examine cortisol concentrations in dogs.

Given the inconsistent findings from previous research on mammals, we did not have strong a priori hypotheses about sex-related differences in HCC among our sample of dogs. Our bivariate analysis indicates that females show higher cortisol than males, but this effect is weaker and inconclusive in models with other predictors. Given the broader literature on the effects of body size on HCC levels, we suspect that the sexual dimorphism of dogs explains this result. As an area for future research, though, the literature would benefit from studies that examine the mechanisms behind sex-related variation in HCC. One possible factor that was not considered in our models is the reproductive status of the female dogs (29). Energetic stress on females during pregnancy is high and continues to be elevated during lactation due to the continued nutritional demand from offspring. To mitigate a potential omitted variable bias, future studies will ideally control for reproductive status.

As with sex, previous findings on cortisol levels and age in canines have proven inconsistent and we did not have a strong prediction as to the link between cortisol and age in our sample population of dogs. Previous findings on cortisol analysis in canine saliva have yielded results indicating juvenile dogs to have significantly differing cortisol levels from adults (18, 19). However, our study indicated no significant difference between juvenile and adult age classes in the Bosawas dog population. These contrasting findings to the prior studies cited may be due to the difference in cortisol analysis methods, as saliva is useful for short-term cortisol analysis whereas hair is beneficial when analyzing cortisol over a period of months.

Partly owing to high levels of malnutrition at Bosawas, we hypothesized that BCS would be negatively associated with the dogs' body condition. The sampled dogs tend to be malnourished, and HCC increases were evident among the dogs in particularly poor condition. This effect remains prominent in a model that includes fur color and other predictors. These findings support our hypothesis that BCS would be negatively correlated with HCC and are congruent with those of prior studies and publications on the energetics of stress. For future research, it would be valuable to determine if this effect remains robust using other methods to operationalize nutritional status.

Prior literature has noted a negative correlation within multiple species between size and cortisol levels where cortisol levels decrease as size increases (5). Therefore, we hypothesized that the geometric mean of morphometric measurements taken from the sampled dogs in Nicaragua would exhibit a negative relationship with HCC. As predicted, our bivariate model indicated that HCC was negatively associated with body size. This effect was weaker and inconclusive in the full model, which again potentially relates to the collinearity between sex, and body size.

More generally, as a preliminary exploration of how cortisol levels may relate to the environmental differences that distinguish our sample, the results from the dogs in Bosawas were compared to those from previous studies on hair cortisol in dogs. Table 3 reports the summary statistics from two prior studies in Switzerland and Italy (10, 30) and compares them with those from our study.

**Table 3:** Comparison of HCC results from the dogs from this study of the dog population in the Bosawas Reserve and prior studies.

**Study**	***N***	**Range (pg/mg)**	**Std Dev. (pg/mg)**	**Mean (pg/mg)**
Bennett and Hayssen (30)	47	4.56–27.09	5.45	12.63
Roth et al. (10)	94	~6–54	(N/A)	~15
Bosawas Population	672	6.1–121.2	9.63	17.88

The means of the three studies did not differ substantially, and all remained between 10 and 20 pg/mg of hair cortisol. However, the range of HCC in our study was much greater than that of the studies conducted above—over four times as high as the maximum HCC reported by Bennett and Hayssen (30), and twice as high as the maximum reported by Roth et al. (10). In addition to the larger sample size in our study, the differences in reported cortisol concentrations between the Roth et al. (10) study and our study may be due to differences in assay methods (radioimmunoassay vs. enzyme immunoassay), but we note that our methodology very closely followed that of Bennett and Hayssen (30). Therefore, a possible explanation is that the wider range of cortisol concentrations reported in the dogs in Bosawas is due to greater prevalence of physiological or environmental stress experienced by the population in comparison to those of the previous studies on healthy dogs.

The similarity in HCC results between the dogs at Bosawas and those from previous studies might initially seem contradictory but could be due to chronic stress experienced by the Bosawas population. Under extended stress, adrenal fatigue occurs, and the body cannot continue to produce high levels of cortisol (2–4). This makes it difficult to use basal-cortisol levels as an indicator when assessing differences between healthy and chronically stressed populations. In addition, during periods of chronic hypocortisolism, the body's response to a stressor is greatly reduced. Accordingly, it may be more valuable to study the short-term cortisol changes in response to a stressor when assessing overall differences between populations (e.g., through sampling feces or urine). In such studies, populations under higher stress on average would likely show less reactivity to a stressor than a population which does not experience chronic stress.

The findings of this study provide further evidence supporting the link between average cortisol levels within the body and variables related to size and nutritional health. Although the dogs at Bosawas were in considerably poorer health relative to those from previous studies on healthy dogs, relationships between cortisol and metabolic rate still appeared in line with previously observed patterns. The inverse relationship between BCS and HCC levels further supports current understandings regarding the interaction of cortisol with nutritional factors in mammals while expanding it to include domestic canids. Among other considerations, this finding may have implications for further research regarding human domestication of dogs and selective breeding (54). Understanding the possible impacts of human selection for size in dogs may enable a better understanding of the physiological processes at work and the ways in which these changes increase the fitness of dogs.

In conclusion, this work contributes to a growing literature on cortisol levels in animal populations, as assessed via hair samples (29). Our results for fur color and nutritional status are consistent with previous findings. This study also suggests important considerations for future research. Previous work suggests that hair cortisol may vary as a function of variables that were not included in our analysis, such as reproductive status, and the season of year (10, 29). In this tropical setting, there are comparatively modest changes in daylight length and temperature. However, there are pronounced differences in rainfall between the rainy season, when these samples were obtained, and the dry season from January to May (55). It would be worthwhile to investigate how this seasonality impacts activity levels and nutritional status, which could in turn impact HCC. More generally, cortisol levels in dogs have been shown to be responsive to social stressors and related aspects of their day-to-day lives (9, 56). In general, dogs in the Bosawas Reserve are typically allowed to roam freely around the community, and they often accompany their owners on excursions to hunt or work in the horticultural plots. However, the Mayangna are rarely observed to pet their adult dogs or interact with them much outside of rebukes for poor behavior. Investigating variation in cortisol as a function of heterogeneity in activities or contacts with others would be a valuable complement to analogous research in settings that bear little resemblance to the Bosawas Reserve. Despite the logistical challenges of conducting this research, cross-cultural studies have considerable potential to elucidate overlooked aspects of human-dog relationships.

## Data Availability Statement

The datasets presented in this study can be found in online repositories. The names of the repository/repositories and accession number(s) can be found at: All data are available as supplemental files on the Open Science Framework: https://osf.io/z7wh9/.

## Ethics Statement

The animal study was reviewed and approved by Institutional Animal Care and Use Committee of University of California, Davis. Written informed consent for participation was not obtained from the owners because Informed consent was obtained verbally as many of the dog owners were functionally illiterate.

## Author Contributions

JK, CF, CS, JG, and RB designed the study. CF, MB-W, JL, LS, AC, DS, JB, JP, AG, MQ, KM, and AA participated in data collection. GB and RB performed sample analysis. MB-W reviewed and organized data. GB and JK performed data analysis. GB, JK, and RB participated in manuscript preparation. All authors contributed to the article and approved the submitted version.

## Conflict of Interest

The authors declare that the research was conducted in the absence of any commercial or financial relationships that could be construed as a potential conflict of interest.

## Footnotes

^1^As an alternative measure of body size, we conducted a factor analysis of the morphometric measurements and regressed HCC on the first factor scores. The inferences were largely comparable to the present use of the geometric mean, though there is evidence that the morphometric measurements do not lend themselves to a single latent factor.
