# Supplementary material for: Fur Color and Nutritional Status Predict Hair Cortisol Concentrations of Dogs in Nicaragua
Source: Front Vet Sci. 2020 Oct 19;7:565346. doi: 10.3389/fvets.2020.565346 (PMC7604343; doi:10.3389/fvets.2020.565346)
Supplement: Supplementary file 1 [file Data_Sheet_1.docx]

Supplementary Material


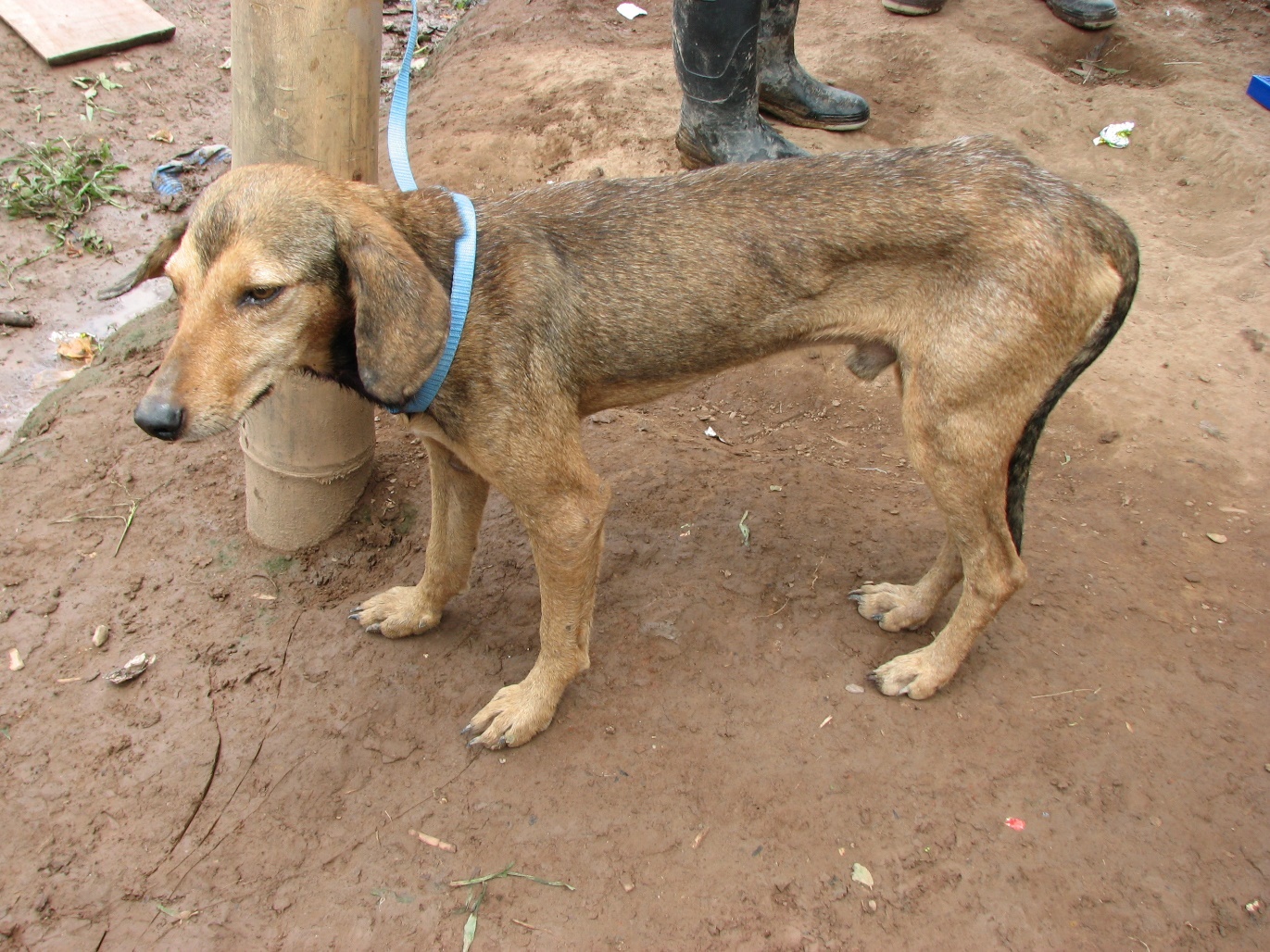


Supplementary Figure 1. Hunting dog from the Bosawas Biosphere Reserve, Nicaragua which participated in our study. Coat coloration classified as mixed (M).


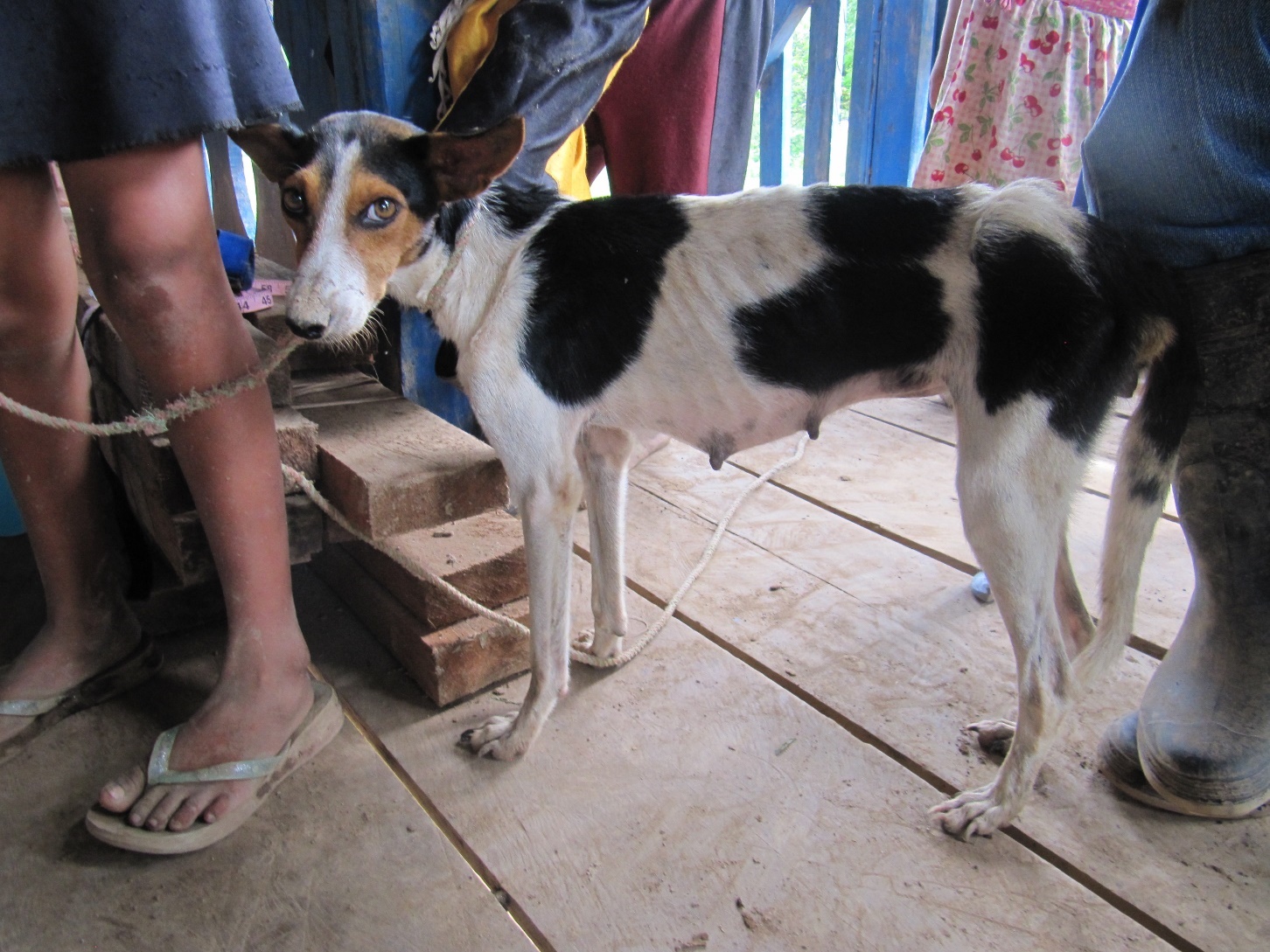


**Supplementary Figure 2.** Hunting dog from the Bosawas Biosphere Reserve, Nicaragua which participated in our study. Coat coloration classified as dark (D) and light (L). Because the fur from the dorsal base of the tail is uniformly dark, this dog would be classified as dark (D) in this analysis.


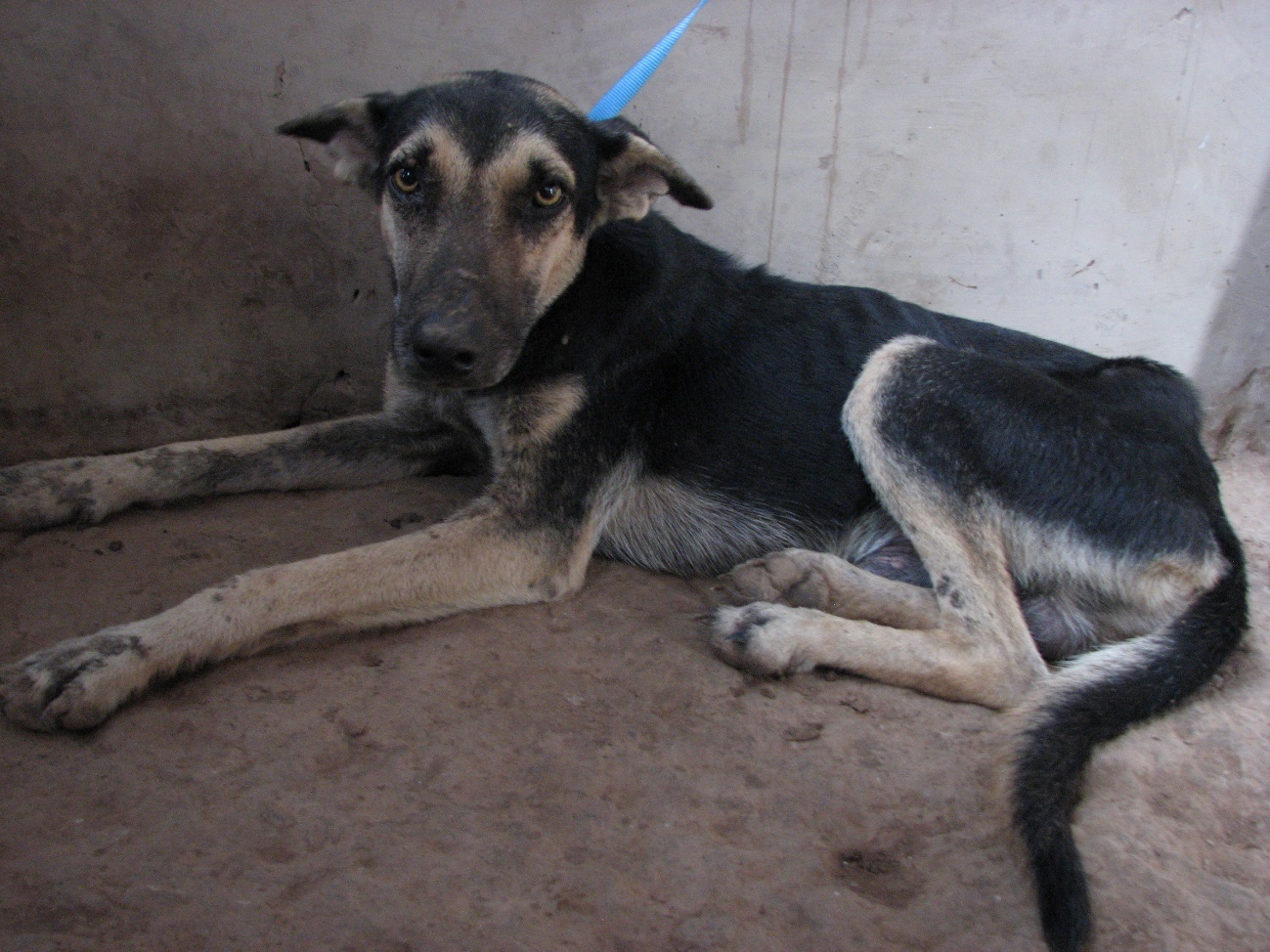


**Supplementary Figure 3.** Hunting dog from the Bosawas Biosphere Reserve, Nicaragua which participated in our study. Coat coloration classified as dark (D) and mixed (M). Because the fur from the dorsal base of the tail is uniformly dark, this dog would be classified as dark (D) in this analysis.


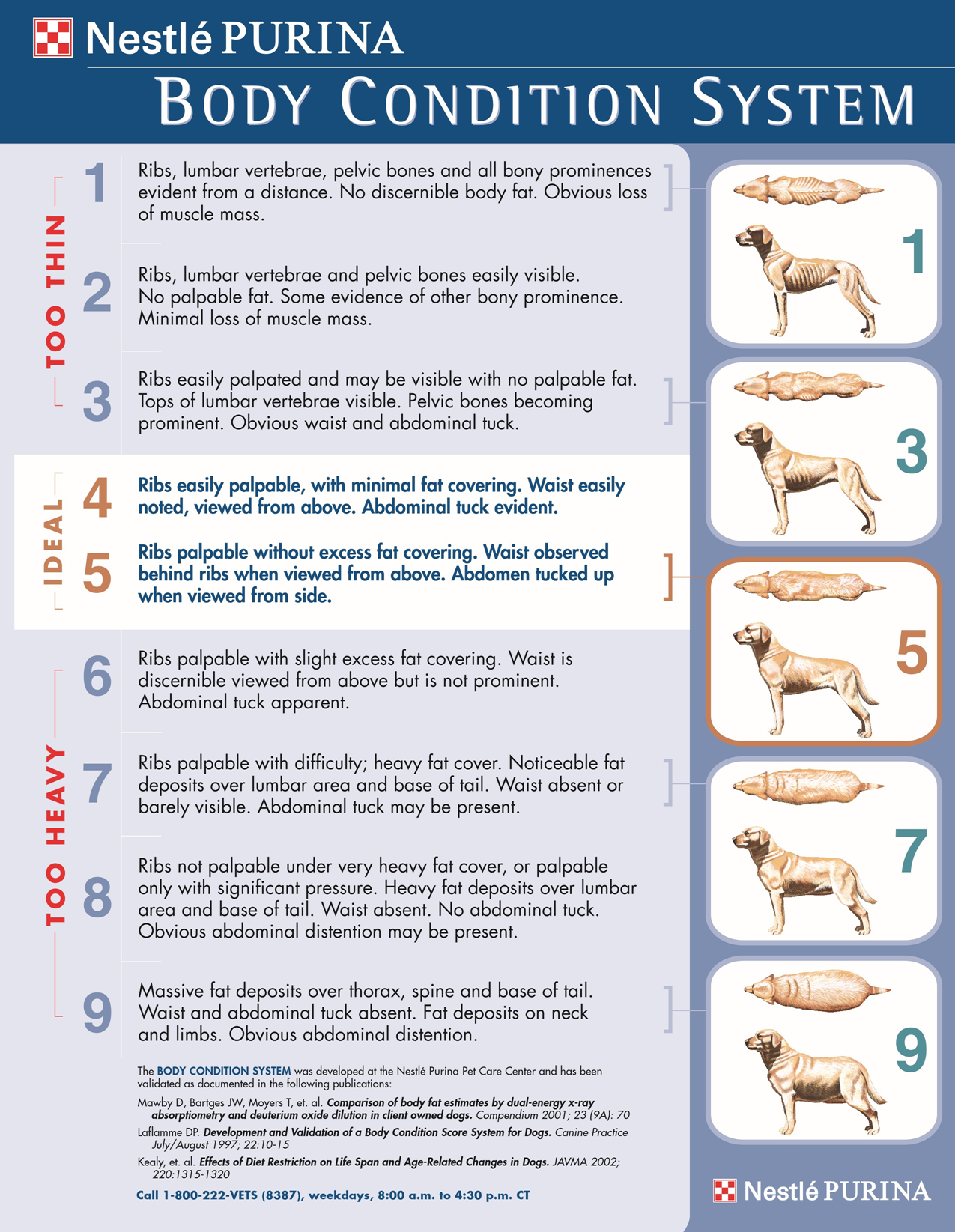


**Supplementary Figure 4.** Body Condition Scoring chart.

**Supplementary Figure 6.** Box and whisker plot of HCC by fur color. Note the untransformed scale of the vertical axis.

**Supplementary Figure 5.** Box and whisker plot of HCC by sex. Note the untransformed scale of the vertical axis.

**Supplementary Figure 7.** Scatter plot of HCC by BCS with fitted trendline for mean HCC by body condition score. Note the untransformed scale of the vertical axis.
